# The Role of Care Coordinator for Children with Complex Care Needs: A Systematic Review

**DOI:** 10.5334/ijic.2250

**Published:** 2016-05-31

**Authors:** Rowan Hillis, Maria Brenner, Philip J Larkin, Des Cawley, Michael Connolly

**Affiliations:** Research Assistant, School of Nursing, Midwifery and Health Systems, UCD College of Health Sciences, Belfield, Dublin 4; Lecturer, School of Nursing, Midwifery and Health Systems, UCD College of Health Sciences, Belfield, Dublin 4; Professor of Clinical Nursing (Palliative Care), Chair, All-Ireland Institute of Hospice and Palliative Care, UCD School of Nursing, Midwifery and Health Systems and Our Lady’s Hospice & Care Services, UCD College of Health Sciences, Belfield, Dublin 4; Lecturer in Nursing Studies, Department of Nursing and Health Science, Athlone Institute of Technology, Dublin Road, Athlone, Co Westmeath; Lecturer, UCD School of Nursing, Midwifery and Health Systems, UCD College of Health Sciences, Belfield, Dublin 4

**Keywords:** care coordinator, complex care, child health

## Abstract

**Introduction:**

This systematic review seeks to identify the intended components of the role of care coordinator for children with complex care needs and the factors that determine its composition in practice.

**Theory and methods:**

The initial search identified 1,157 articles, of which 37 met the inclusion criteria. They were quality assessed using the SIGN hierarchy of evidence structure.

**Results:**

Core components of the role include: coordination of care needs, planning and assessment, specialist support, emotional support, administration and logistics and continuing professional development. Influencing factors on the role include the external environment (political and socio-economic), the internal environment (organisational structure and funding protocols), the skills, qualifications and experience of the coordinator, the family circumstances and the nature of the interaction between the care coordinator and the family.

**Discussion:**

The lack of consistent terminology creates challenges and there is a need for greater consensus on this issue. Organisations and healthcare professionals need to recognise the extent to which contextual factors influence the role of a care coordinator in practice and plan accordingly. Despite evidence that suggests that the role is pivotal in ensuring that care needs are sustained, there remains great variability in the understanding of the role of a care coordinator for this population.

**Conclusions:**

As the provision of care increasingly moves closer to home there is a need for greater understanding of the nature and composition of the interaction between care coordinators and families to determine the extent to which appropriate services are being provided. Further work in this area should take into consideration any potential variance in service provision, for example any potential inequity arising due to geographic location. It is also imperative, where appropriate, to seek the views of children with complex care needs and their siblings about their experiences.

## Introduction

The provision of care closer to home for children with complex care needs is a policy objective internationally [1,2,3]. For many of these children, their reliance on technology demands a tailor-made service to ensure that care within the home is viable and sustainable [4]. Progress towards achievement of this goal has been slow [3] despite growing evidence that homecare: provides a means of mitigating the barriers and isolation children and their families experience during the transition from hospital to home, can significantly reduce hospital utilisation, and reduces the cost of care for children with complex care needs [5,6,7,8,9].

Even with the benefits of homecare, the number of healthcare professionals, care settings and treatments that underpin individual care plans for children with complex care needs represents an often difficult and challenging exercise in care coordination for families [10]. An integrative review of the constituents of complex discharge, drawing from 34 key documents, highlights that “key working (a designated coordinator) is essential for overseeing the entire transition process and ensuring a communication loop across the health system” [11, p. 984]. While the intended role of case managers has been discussed, evaluated and analysed internationally [1,12,13,14], using interchangeable titles such as Family Care Coordinator, Care Coordination Counselor, Nurse Care Coordinator, and Key Worker [15,16,17,18,19,20,21], there is no consensus within the literature internationally on the key constituents of this service in practice.

It is imperative to address the issue of appropriate care coordination for a growing population of children with complex health issues. Recent population prevalence estimates by the World Health Organisation suggest that one in every 33 infants are born with a congenital malformation [22], while in the UK estimates suggest that 32 per 10,000 children under 19 years have a life-limiting condition and ongoing complex needs [23]. Children living with long-term ventilator dependency have increased nearly 10 fold in the UK since 1999, with some geographical areas seeing a 30-fold increase in prevalence since 1994 from 0.2–6.7 per 100,000 [24]. Follow-up studies show that children with tracheostomies and positive pressure ventilation have a 5-year survival rate of 89% once home with 25% being decannulated [25], thereby reinforcing the need to have an adequate care coordination service available to these families.

Due to the paucity of research specifically in this area and the broad variation of job titles known to be in use, the aim of this systematic review was to evaluate and review empirical and theoretical literature, to provide a detailed critical overview of the nature and composition of the role of care coordinator for children with complex care needs. This approach is distinguished from other review methods by its underpinning structured methodology [26,27]. As such, it facilitates presentation of a quality-assessed evidence-based perspective on the role of a care coordinator in practice. The objectives of the review were to identify published accounts of the role of care coordinators; to carry out a quality appraisal of such accounts; to present a conceptual model that contextualises the role of care coordinator within the wider health service and to highlight the nature and composition of the role in practice.

## Theory and Methods

The broad variation in job titles and environments in which care coordinators work, required a review process of sufficient breadth to encapsulate all aspects of the role and enough depth to provide the focus required to inform practice for children with complex care needs. The methodology was guided by a five stage process [26] which has been recognised for its rigour [28] and used across a range of nursing specialties [29,30]. The five stages are: problem identification, literature search, data evaluation, data analysis, and presentation of results.

### Problem Identification

Problem identification involves specifying variables of interest (concept, target population and clinical area [26]). For the purposes of this review, the problem was identified as examining the nature and composition of the role of care coordinator (concept) for children (target population) with complex care needs (clinical area).

### Literature Search

Reflecting best practice, the search strategy sought to identify literature from three perspectives, [26,27]. Conducted in February 2015, the first perspective identified and explored three bibliographic databases: CINHAL, Medline via PubMed and PsycInfo. In addition to key word searches, related MeSH terms, MedLine Theasaurus and CINAHL Headings were added to the search criteria and Boolean terms applied accordingly. Keywords ‘care coordination’ and ‘care coordinator’ were used to ensure that all potentially relevant articles were encapsulated (Table 1).

**Table 1:** List of Keywords/Mesh Terms.

Key Terms: Role/Process	Medline Via Pubmed Mesh Term	Cinahl Plus – Cinahl Headings	Proquest Psycinfo – Thesaurus

Case manager / managers / management	Case management	Case Managers	n/a
Case worker / workers / working	Social Work	n/a	n/a
Key worker / workers / working	n/a	n/a	n/a
Care coordinator / coordinators / care coordination	n/a	nursing care coordination	n/a
Nursing care coordinator / nursing care coordinators / nursing care coordination	n/a	nursing care coordination	n/a
Service Manager / managers / management	n/a	n/a	n/a
multi-agency working / multidisciplinary team	n/a	multidisciplinary care team	

**Key Terms: Patient Group**			

Children	Child, disabled children, hospitalized child	Child medically fragile, child disabled	n/a
Child	Child, hospitalized child,	Child disabled, child medically fragile	n/a
Paediatric / pediatric / paediatrics / paediatrics	Pediatrics	Pediatric care	Pediatrics
Young adult / young adults	Young Adult	Young adult	n/a
Youth / youths	Adolescent	Adolescence	n/a
Adolescent / adolescents	Adolescent, adolescent health services, hospitalized adolescent	Adolescent hospitalised / adolescent hospitalized	n/a
Young people	n/a	young adult	n/a
Young person / young persons	n/a	young adult	n/a

**Key Terms: Context**			

Complex care	Tertiary Healthcare	Tertiary health care, multidisciplinary team	n/a
Disability	Disability evaluation, Disabled children, health services for persons with disabilities, chronic disease	Disability	n/a
Intellectual Disability / intellectual disabilities	Intellectual Disability		
Disabled	Disabled children, health services for persons with disabilities, child health services, adolescent health services, health services needs and demands	Disabled	n/a
Chronic care	Long-term care	Multidisciplinary care team	n/a
Home telehealth	n/a	home health aides, home rehabilitation, home health care	n/a
Special health care needs	n/a	health services needs and demand, needs assessment	n/a
Medical complexity	n/a	n/a	n/a
Palliative care	Palliative care, hospice and palliative care nursing	Palliative care, hospice and palliative nursing, multidisciplinary care team, health services needs and demand	Palliative care

**Figure 1:**
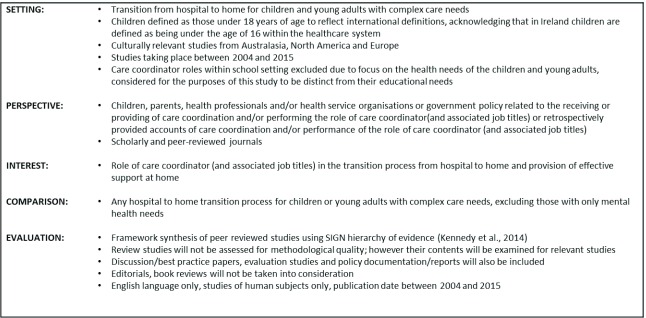
SPICE Framework and inclusion criteria.

Literature identified was subjected to ancestry and citation index searching to identify any further studies of relevance. Articles included were limited to peer-reviewed quantitative, qualitative and mixed method articles, which were published in English between 2004 and 2015. This 11 year time frame was applied to reflect the contemporary nature of the problem under review and articles identified in English from Europe, North America and Australasia were considered to be culturally closest to the Irish context. The second perspective involved journal hand searching, a search to identify relevant conference proceedings and requests for additional references from the team’s network of geographically dispersed researchers in this area [31]. The third perspective involved a generic internet search using Google Scholar, which also sought to identify other grey literature in the area. The SPICE Framework was adapted and applied to facilitate parameter definition and inclusion criteria (Figure 1). We considered a number of frameworks for this purpose, to enable the development of a clearly defined and well-structured review, including PICO and ECLIPSE. The emphasis on persective in this framework was particularly important as we were exploring a role that is a key axis in the interace of primary and secondary care.

**Figure 2:**
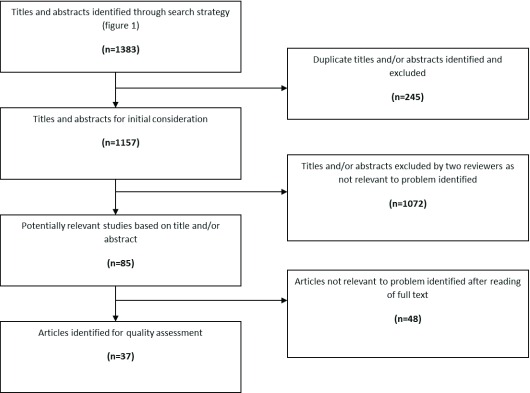
Search Result.

The combined search resulted in 1,343 articles, of which 186 were identified as duplicates, leaving 1,157 articles for initial consideration. The abstracts were assessed against inclusion/exclusion criteria (Figure 2), 1,072 of which were then excluded. A further 48 were excluded after reading the full text, where they did not pertain specifically to care coordination, referred to the wrong patient group and where the outcomes were not of relevance to the area of care coordination. This left a sample of 37 articles to be quality assessed. It is acknowledged that despite efforts to the contrary, it is possible that papers in press or grey literature may have been missed during the course of the search. Of the 37 articles considered, 25 were based on empirical research. Of those, nine focused specifically on the staff perspective, seven on family views, seven on comparing and contrasting the views of staff, families and/or stakeholders, and three examined documentary evidence of activities undertaken. The remainder comprised of literature reviews, guidelines or policy recommendations. Although aspects of the children’s illnesses were examined, no study set out to capture the views of children with complex care needs. Sample sizes varied from single case studies [15,32] to studies with over 200 respondents where quantitative data collection techniques were used.

### Data Evaluation

The insights offered by methodologically robust studies were considered to offer the most credible and valid insights into the role of care coordinator. The updated Scottish Intercollegiate Guidelines Network (SIGN) hierarchy of evidence [33] was used to assess the strength of evidence upon which each study is based. This criteria is widely used in the literature to assess the quality of studies for inclusion in systematic reviews [34,35,36]. It offers a clear grading structure of level of evidence as follows: 1++ = high quality meta analysis, systematic reviews of RCTs, or RCTs with a very low risk of bias; 1+ = well conducted meta analyses, systematic reviews, or RCTs with a low risk of bias; 2+ = well conducted case control or cohort studies with a low risk of confounding or bias and a moderate probability that the relationship is causal; 3 = non-analytic studies; and 4 = expert opinion. The articles were grouped according to methodological approach: quantitative, qualitative, mixed method and reviews. Four reviewers worked in pairs, examining two groups each. Reviewers (MB, PL, DC, MC) independently assessed each article and then compared and discussed their findings with the other reviewer assigned to their group. The presence of potential bias is acknowledged as reviewers were not blinded to study identifiers such as author name, institution or journal [37]. Differences in opinion on gradings occurred for four papers and this was resolved through discussion. Of the 26 studies, one [8] was graded as a 2+, 17 studies were graded as 3, (non-analytical studies), and eight were graded as 4 (expert opinion). In addition to the 26 empirical studies, three literature reviews received a rating of 3 [38,39,40]. The remaining eight articles were not considered to have sufficient evidentiary support.

### Data Analysis

To enable rigorous, consistent, data abstraction and synthesis, the team extracted key data and findings into a pre-formatted table. This facilitated constant comparison and identification of emergent themes and followed best practice [26]. A standard template was designed to summarise descriptive information (author name(s), article title, publication date and study origin), methodological data (setting, sample size, study design and data collection methods), key messages (study aim, key themes, limitations) and outcome of the SIGN quality assessment (Table 2).

**Table 2:** Summary of articles reviewed.

Author	Title	Approach	Sample Size	Empirical Research Perspective	Key Focus	Origin	Conclusions

Albanesi *et al.* (2009)	Role of disability-case manager for chronic diseases: Using the ICF as a practical background	Case Study	Single Case	Staff	Identifies how case managers contribute to care of children with disabilities	Italy	The case manager’s role is fundamental to support patients and their families; and one of its key interventions is the creation of a network around a person with complex care needs where this network does not exist.
Howitt (2011)	The family care coordinator: Paving the way to seamless care	Case study	Single case	Staff	Illustrate the concept of family care coordination through case study	Canada	Key components of the role are ongoing assessment, education, partnerships, communication, support and advocacy. Essential resources and pathways are required to implement the role and optimise outcomes. Challenges are identified to include time constraints, maintenance of boundaries and emotional burnout.
Care Coordination Network UK (2009)	Care Coordination Network UK: Key Worker Standards	Guidelines			Sets out guidelines for key worker standards	UK	
Mengoni et al., (2014)	Developing Key Working	Guidelines			Offering guidelines to those developing key working services	UK	
Department of Health (2009)	Integrated Care Pathway for Children and Young People with Complex Physical Healthcare Needs	Integrated Care Guidelines			Guide for community services in meeting the needs of families, children and young people, aged up to 18 years, who have complex physical healthcare needs	UK	
Fraser et al. (2009)	Factors that influence case managers’ resource allocation decisions in pediatric homecare: An ethnographic study	Qualitative - Interviews, card sorts, participant observation over a 5 month period	11 case managers	Staff	Factors that influence decision making by nurse case managers	Canada	The study provides new insights into resource allocation decision-making, offering a taxonomy to identify and classify influencing factors.
Ehrlich et al. (2009)	Coordinated care: what does that really mean?	Literature review			Identifies and examines the core attributes of care coordination within the primary care context	Australia	Offers a framework of coordinated care within the primary care setting that takes into consideration the key attributes of coordinated care that were identified during the review, with the aim of guiding future work around implementation and evaluation.
King and Meyer (2006)	Service integration and co-ordination: a framework of approaches for the delivery of co-ordinated care to children with disabilities and their families	Literature review			Aims to provide clarity and direction to provision of coordinated care	Canada	Offers a framework that can be used to support policy- and decision-making in the context of co-ordinated care provision.
McSpadden et al, (2012)	Care coordination for children with special health care needs and roles for physical therapists	Literature review			Summarise benefits of care coordination and explore potential roles for physical therapists	USA	Therapists need to be aware of and adapt to change in care models in order to be the provider of choice.
Robinson (2010)	Care coordination: a priority for health reform	Literature review			Outlines policy recommendations needed to enhance care coordination	USA	Recommendations include the need to facilitate better information transfer with wider use of information technology, include nurse practitioners as equal practitioners in reimbursement, create incentives to improve care coordination, reward the use of evidence based practice and advocate for better care coordination models
Greco et al. (2005)	An Exploration of Different Models of Multi-Agency Partnerships in Key Worker Services for Disabled Children: Effectiveness and Costs	Mixed methods	225 Children with Disabilities Teams, 70% response rate, 87 interviews with key workers, questionnaires by 205 parents and 30 children	Multiple	Compare models of key working, identify areas for best practice, investigate sources of funding	UK	Key workers provide a valuable service that has a positive impact on many families’ lives and their collaborative approach facilitated access to appropriate support. However, outcomes vary across different areas, dependent on service management, understanding of the role and provision of training and supervision
Rahi et al. (2004)	Meeting the needs of parents around the time of diagnosis of disability among their children: evaluation of a novel program for information, support, and liaison by key workers	Mixed methods	79 families from pre-group and 68 from post (68% and 65% response)	Family	Care coordination needs at time of diagnosis	UK	The greatest needs during the critical period around diagnosis are for information as well as emotional support from professionals, informal & formal networks and support groups.
Rodriguez and King (2014)	Sharing the care: the key-working experiences of professionals and the parents of life-limited children	Mixed methods	35 at focus group, 25 interview	Multiple	Exploring the lived experience of caring and care planning for children with life limiting conditions	UK	The findings are limited by sample characteristics however they provide insight for current policy & practice initiatives. Key works need to be mindful of historic care arrangements and be prepared to step into the ‘family team’ arrangements.
Taylor (2012)	Implementing a care coordination program for children with special healthcare needs: partnering with families and providers	Mixed methods	91 patients under care of care coordination counsellor, 439 patients provided with care binder	Multiple	Service evaluation, evaluating impact of care coordination counsellor service	USA	Patients supported by the counsellor service reported greater agreement when accessing resources and identifying a key point person for coordination.
Wood et al. (2009)	A multi-method assessment of satisfaction with services in the Medical Home by parents of children and youth with special health care needs (CYSHCN)	Mixed Methods	6 practices, 262 (75% response) families completed questionnaire, 28 families in focus groups	Family	Assess satisfaction of parents with treatment by office staff, communication with the paediatricians, involvement in decision making and coordination of services outside the practice	USA	Paediatricians must become better equipped to identify and communicate more proactively with parents of children with CYSHCN who are under significant stress; and they and their staff must also improve their knowledge of community resources.
Webb et al. (2008)	Key workers and schools: meeting the needs of children and young people with disabilities	Mixed methods – interviews 7 service managers, 32 steering group members and 50 key workers, questionnaires completed by 189 parents and subset of 68 parents for interview	7 case study areas	Multiple	Relationship between key worker services to promote inter agency care coordination and schools	UK	Key workers can improve home-school relationships, facilitate the contribution of teachers in inter-agency working, enable mainstream schools to better meet the needs of pupils with disabilities and improve their inclusive practice.
Beecham et al. (2007)	The costs of key worker support for disabled children and their families	Mixed methods – interviews and questionnaire	7 service sites	Multiple	Identifies costs associated with providing care coordination services	UK	The low response rate and absence of data on some elements impacts generalisation of findings. Their findings highlight that contact costs varied depending on level of disability and number of role aspects performed by the key worker.
Cady et al. (2014)	Attributes of Advanced Practice Registered Nurse Care Coordination for Children With Medical Complexity	Mixed methods – interviews, documentary analysis, survey	2628 care coordination episodes conducted by telehealth over consecutive 3 year time period for 27 children	Multiple	Investigates attributes of relationship-based advanced practice registered nurse care coordination	USA	The advanced practice registered nurse care coordination model has potential for changing the health management processes for children with medical complexity.
Purves et al. (2008)	The development of care coordination services in Scotland: A report to Care Co-ordination Network UK	Mixed methods, questionnaire to all 32 Scottish local authorities, telephone interviews	22 questionnaires returned (69% response)	Staff	Demonstrates extent to which progress has been made in Scotland since 2004 and highlights where further work is needed	UK	There are number of challenges facing care coordination services including: funding issues, ongoing challenges of interagency working, qualification criteria, proliferation of coordinated planning mechanisms, providing family & child-centred services, understanding of the key worker’s role, the training and the development of key workers
Fitzgibbons et al. (2009)	Care management for children with special needs: Part II: the role of primary care	Documentary analysis	2 Primary care practices in 5 counties in Washington state	Patients (children 17) with or at risk of a chronic condition as per Clinical Risk Groups Software. 189 initially selected (final sample 161)	Documents care management services	USA	Paediatric clinical care management activities directly relate to patient care and are complementary to, not duplicative of, case management provided by health plan managers.
Carter et al. (2007)	An exploration of best practice in multi-agency working and the experiences of families of children with complex health needs. What works well and what needs to be done to improve practice for the future?	Qualitative – appreciative interviews, nominal group workshops and consensus workshops	20 mothers, 7 fathers, 1 child, 41 working with children	Multiple	Discusses what works well, why it has worked well and what best practice in the future could be	UK	The results suggest that parents need the opportunity to share and receive support from other parents who understand the reality of caring for a child with complex needs. Collaborative working needs to underpin the appointment of the most appropriate person to act as long-term coordinator where required by families.
Golden and Nageswaran (2012)	Caregiver voices: coordinating care for children with complex chronic conditions	Qualitative – focus group	14 care givers	Family	Explores care givers’ perspective	USA	More information sharing and quality communication is needed among those providing care, caregivers need help in navigating the system of care, and caregivers develop strategies to cope with care coordination demands. The burden of coordinating care can be alleviated in part through improved communication and collaboration.
Ehrlich et al. (2013)	How does care coordination provided by registered nurses “fit” within the organisational processes and professional relationships in the general practice context?	Qualitative – interpretative, using focus groups	9 registered nurses from 5 general practices	Staff	How nurse provided care coordination can fit into organisational processes	Australia	Registered nurse-provided care coordination could ‘fit’ within the context of general practice if it was adequately resourced. Successful development of the role requires attention to educational preparation, support of the individual nurse and attention to organisational structures.
Law et al. (2011)	Managing change in the care of children with complex needs: healthcare providers’ perspectives	Qualitative, semi structured interviews, focus groups, telephone interviews	3 nursing and four allied health managers telephone interviewed, focus groups with 15 nursing and 11 AHP, and 3 nurses and 1 speech therapist interviewed by phone	Staff	Description of the role and activities of nursing and AHP caring for children with complex needs in a community setting	UK	Findings support the adoption of integrated partnership working, going beyond the identification of key professionals, to developing a set of criteria against which future service provisions could be judged.
Kingsnorth et al. (2015)	Inter-organizational partnership for children with medical complexity: The integrated complex care model	Qualitative, semi-structured interviews, focus groups, document review and audit of administrative databases	12 families, 10 committee members, 7 key workers, 4 healthcare professionals - 21 in total for focus groups	Multiple	Identification of areas where care coordination can be improved at a systems level	Canada	At a systems level the integrated model fostered collaboration between partner organisations. At family level, development of inter-organisational management structures and communication platforms, provision of adequate resourcing, and increased engagement of primary care may enable high level organisational integration aimed at improved care coordination.
Brustrom et al. (2012)	Care Coordination in the Spina Bifida Clinic Setting: Current Practice and Future Directions	Semi structured interviews with clinic staff, focus groups with care givers	43 staff, 38 caregivers through focus groups	Multiple	Examines elements of care coordination in spina bifida clinic setting	USA	Study findings suggest ways that care might be coordinated optimally in spina bifida clinics. A synthesis of these findings for clinics interested in implementing care coordination or improving the care coordination services they currently offer is provided.
Sloper et al. (2006)	Key worker services for disabled children: What characteristics of services lead to better outcomes for children and families?	Quantitative	189 parents across 7 key worker schemes	Family	Examines which aspects of key worker schemes are related to better outcomes for families	UK	There is a need for regular training, supervision and peer support for key workers and negotiated time and resources for them to carry out the role. These influence the extent to which key workers can carry out aspects of the role and their amount of contact with families, which in turn impacts outcomes.
Palfrey et al. (2004)	The pediatric alliance for coordinated care: evaluation of a medical home model	Quantitative - completion of survey at baseline and follow up at 2 years.	150 children with complex needs from 6 practices	Family	Determine satisfaction with care coordination intervention	USA	The PACC medical home intervention increases parent satisfaction with pediatric primary care. Those whose needs are most severe seem to benefit most from the intervention. There are some indications of improved health as well as decreased burden of disease with the intervention in place.
Antonelli et al. (2008)	Care coordination for children and youth with special health care needs: a descriptive, multisite study of activities, personnel costs, and outcomes	Quantitative – document analysis. Adaptation of the University of Massachusetts Medical School Care Coordination Measurement Tool	6 general paediatric practices	Documentary analysis	Examines activities carried out by care coordinators and costs associated with role of care coordinator	USA	The presence of acute, family-based social stressors was a significant driver of need for care coordination activities. A high proportion of dependence on care coordination performed by physicians led to increase costs. Office-based nurses providing care coordination were responsible for a significant number of episodes of avoidance of higher cost use outcomes.
Greco and Sloper (2004)	Care co-ordination and key worker schemes for disabled children: Results of a UK-wide survey	Quantitative, postal survey	225 Children with Disabilities Teams, 70% response rate	Staff	Explore the nature and variation of care coordination services	UK	The proportion of areas having care coordination or key worker services is consistent with findings on research with parents of disabled children. The extent of multiagency involvement in planning and overseeing the operation of the service was positive but joint funding was more problematic. There was considerable variation in service models.
Park et al. (2009)	The Evidence Base for Case Management Practice	Quantitative, secondary analysis	4,419 case managers responding to online survey conducted by Commission for Case Manager Certification	Activity analysis	Compare case management activities and knowledge elements by profession and work setting	USA	There is evidence for how to develop case management programs consistent with both organisational characteristics and strengths of the nursing profession.
Petitgout et al. (2013)	Development of a hospital-based care coordination program for children with special health care needs	Service Evaluation			Describes the development of a hospital based inter-professional care coordination program for children with complex care needs	USA	Pediatric nurse practitioners play an important role in the medical home, collaborating with primary care providers, hospital-based specialists, community services, and social workers to provide services to children with special health care needs.

## Results

The main theme that emerged from the literature is that the composition of the role of the care coordinator depends upon an infinite number of contextual variables. The core elements of the intended role are consistently identified within the literature but the variables that determine the actual composition of the role appear numerous and unpredictable.

### Intended Tasks and Activities

Sixteen papers reviewed discussed the range of tasks and activities intended to be undertaken by care coordinators: two quantitative studies [41,42], two qualitative studies [4,43], three literature reviews [36,37,44], three guidelines [12,45,46], four mixed methods studies [16,19,38,47] and two case studies [15,32]. Although the models of health systems differ internationally the core expectations of the activities carried out by care coordinators are consistent. Findings from these papers highlight the intended role of care coordinator as being one which encompasses tasks under four key headings: coordination of care needs, planning and assessment, information and specialist support, and emotional and practical support [12]. Coordination of care needs can include improving access, navigating the complexities of multiple service providers, and/or establishing service provision links [32,38,44,48,49] as well as functioning in a problem solving capacity as required [32]. Planning and assessment includes the coordination of future visits, studies or referrals [44,47,49], ensuring treatment plans are carried out [47,49], and the initial and ongoing identification of needs [15,36,37,49]. Information and specialist support involves the knowledge and provision of clinical and local information [39,45,48], acting as a point of reference for all enquiries related to the client [36] and sharing information with professionals and families [49]. Emotional and practical support refers to the provision of support in a crisis [48], making travel and education arrangements, accompaniment and support during hospital visits [32] and speaking on behalf of families in an advocacy capacity as required [45,48].

In addition to the role performed, four articles [4,16,19,42] highlight three other key elements inherent within the role: administration and logistics, self-care, and continuing professional development. Administration and logistics refers to the writing of case notes, travelling between clients, liaising with colleagues, attending internal meetings [19] and dealing with the bureaucracy involved in obtaining funding for specific pieces of equipment [4]. Such findings emerged during the course of focus groups [4], interviews with staff [19] and a questionnaire specifically designed to examine care coordination activity [42].

### Intended Nature and Composition

Four articles highlighted the expected composition of the role of care coordinators: two activity tracking studies [13,42] and two qualitative studies, one with staff [50] and one with both staff and families [51]. Collectively they found that care coordinators are expected to have multiple skills, share a common vision, have a mutual respect for each other’s role when dealing with the family, have the freedom to be innovative and work collaboratively, be able to function independently and work autonomously, manage time effectively, build networks, create and maintain relationships and problem solve when required.

### Influencing Factors

Fraser et al.’s ethnographic study of the influences affecting resource allocation decisions draws attention to the difficulty that case managers have in categorising the influencing factors; “The words ‘it depends’ became a mantra” [52, p. 345]. This ‘mantra’ is omnipresent throughout the literature, with four key variables identified as impacting upon the actual composition of the role: the economic, political, socio-cultural, legal and technological environment (external environment) in which the health service provider operates; the structure, size and funding of the health service provider (internal environment); the individual skills and experience of the care coordinator (the individual); and the nature of the relationship and interaction between the care coordinator, the child and the family (the interaction).

#### The external environment

Multiple variables in the external environment are identified in the literature and highlight the fragmented nature of health service provision [32,40,41] and the disparate integration between services and the inequity of service provision [4]. This can lead to care coordinators operating within a specialised sphere of experience, unwilling to meet unmet needs outside their realm of expertise, and to parents being hindered by differences in terminology across service providers [41]. Funding is highlighted as a fundamental factor in service provision [47,50,52], which in turn is influenced by government policy [53].

#### The internal environment

Variables associated with the internal environment fall under two broad headings: structure and resources. Models of care can vary within the same umbrella service provider, particularly in relation to designated or non-designated care models, which have a direct bearing on the caseload of care coordinators [41,54]. The length of time the service provider has been in place can also have a bearing on the range of activities carried out by key workers; the ‘younger’ the service, the more aspects of the role they often undertake [18]. Staffing and equipment are identified as key resource variables. For example, the challenges of recruitment and levels of pay as well as cover during sickness or leave are highlighted in the literature [15,42], there may be differences in levels of communication between disciplines [32,47], while others consider success to be dependent on a leadership that values the skills base of nurses and which places emphasis on the importance of relationships within the team [50,55].

#### The individual

In their secondary analysis of the activities undertaken by case managers from a range of professional backgrounds, Park et al. note that the success of the role is dependent “largely upon case managers’ individual capabilities rather than clearly defined roles and functions” [56, p. 694]. This desirable range of capabilities and qualities is apparent across the literature sample. The range of backgrounds and level of experience held by care coordinators is also identified as a core variable; care coordinators, although predominantly from health visiting, nursing, teaching or social work, can also come from other allied health professional backgrounds, such as occupational or speech therapy [19,52,57]. This variation in skills and experience also impacts upon the level of educational preparation needed by each individual care coordinator, for example there is a need for health professionals working with children to strengthen their knowledge of child and family health [4].

#### The interaction

Four studies identified variations in time spent with the client and family, due to case load and the nature of the model of care (designated or non-designated), as a key variable that impacts the interaction between the care coordinator and the family [15,42,44,54]. In addition, the nature of the interpersonal relationship between the care coordinator and the client and their family varies. For example, an exploration of the lived care experiences of parents and keyworkers dealing with children with complex care needs identified that the professional is there to work with the family and not for the family, which can be a difficult balance to maintain [21]. This is supported in research which refers to the emotional burden and challenges of maintaining professional boundaries when working in such an emotionally charged environment [7,15], while others identify buy-in and a belief in the value of the service on the part of the family respectively as being integral to a successful relationship [55,58]. This may be extended further, in that it is suggested that the variation of parent and family characteristics and expectations may influence the relationship [47,58]. In particular some families expect to be told exactly what to do while others seek mutual consultation and collaborative decision making. Finally, at the crux of the relationship lies the child with complex care needs. The intensity of care needed is dependent upon the complexity of their physical and psychosocial conditions [51,59], which in turn has a direct impact upon the physical and psychosocial condition of the family.

## Conceptual Map of Key Findings

**Figure 3:**
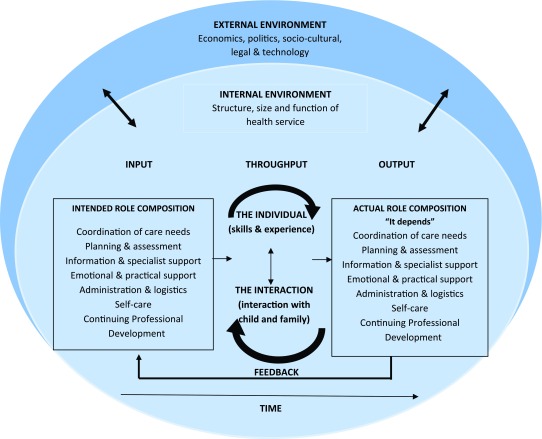
Conceptual map of findings.

Synthesis of the findings reveals the extent to which the actual role of the care coordinator is influenced by a broad range of variables. The conceptual framework (Figure 3) provides a visual representation of the dynamic process that influences the nature and composition of the role in practice. The model draws from general systems theory as a means of contextualising the role of the care coordinator within the wider health system in which children with complex care needs receive care. Katz and Kahn first used general systems theory to examine organisational behaviour as a series of open systems interactions, which are interdependent, consistent over time, cyclical and understood in the context of their interaction with each other and the external environment [60]. Conceptually, this presents as an input-throughput-output-feedback model, illustrating a knowledge framework focused on structure, relationship and interdependence between elements [60]. Input reflects the inflow of energy and information from the external environment, throughput reflects the reorganisation of engergies within the system and output highlights that the product of the interactions must be exported into the external environment. Systems perform as cycles of events and feedback ensures that internal information is used to adjust the intake of energy and information from the external environment [60,61]. Inherent within this approach lies the premise that human beings are also unique open systems that continuously interact with their external environment resulting in a state of constant change that, in health terms, presents in varying degrees of wellness. The model is also underpinned by the universally accepted family-centred care philosophy, which recognises the crucial nature of the family’s involvement in ensuring the health and well-being of child, and demands that healthcare staff work in partnership with caregivers to provide children with a safe and effective care plan [62,63].

## Discussion

The important contribution made by this systematic review is the contextualisation of the role of care coordinators. The infinite number of variables that exert influence over the composition of the role in practice have been inferred but not explicitly discussed in the literature. Policy internationally aspires to ensure that children with complex care needs are cared for in the home, even with increasing technology-based needs. This demands that care plans are tailor-made, placing demands on the structure and funding of community-based care services. Whilst standardised job descriptions offer a means of justifying funding and ensuring cost-effective service provision, such standardisation contradicts the demand for tailor-made care plans. This leads to a number of implications for education and development, research, service delivery and policy.

### Implications for Recruitment, Training and Development

It is essential that organisations and healthcare professionals recognise the extent to which contextual factors influence the role of a care coordinator in practice and plan accordingly. Recruitment practices need to be an integral part of the strategic planning process, ensuring that adequate funding is in place to attract and retain high calibre staff who have the technical and interpersonal skills demanded by the role. As the literature highlights, the core elements of the role are relatively consistent internationally, which allows for generic training and development of staff, however, individual organisations must make provision for context specific education and training to meet the needs of their client population. This is particularly important for care coordinators without a specific healthcare background, to ensure that they have sufficient skills to be able to deliver a safe and effective service for the family. This work also highlights the importance of ongoing development of those in post to ensure that they have the skills necessary to deal with the evolving needs of the children within their care.

### Implications for Policy and Service Delivery

The role of care coordinator is not consistently operationalised in some states internationally. It is important that the roles which do exist are evaluated to understand what is working well and to consider what is necessary to improve. However, the success of such a role is also dependent on the support structures around it. This requires agreement and direction at policy level on the criteria for competent care delivery and clarity in the responsibility and regulation of training and education of nurses and healthcare staff caring for these children.

### Implications for Future Research

Despite the importance families place on having a care coordinator, the lack of consistent terminology creates challenges for rigorous comparisons across various service models. Levels of evidence underpinning research in this area vary, with the majority of studies being categorised as non-analytic studies or expert opinion. While this is to be expected at the exploratory stages of research in an area, as care practices are introduced and refined, research is required to evaluate innovative practices. The literature highlights the broad range of skills that care coordinators are expected to possess, yet little is known as to the extent to which these skills are innate or can be taught. Furthermore, as the provision of care closer to home is increasingly recognised as an objective for care for these children and their families [1,2,3] and a greater demand for tailor-made services for children with complex care needs is now omnipresent, the literature highlights a need for further research into the nature and composition of the interaction between care coordinators and families to determine the extent to which such services are being provided. Such studies must also take into consideration any potential variance in service provision, for example between rural and urban areas, to identify any potential inequity arising due to geographic location. It is also imperative, where appropriate, to seek the views of children with complex care needs and their siblings about their experiences.

### Limitations

A number of interchangeable titles are used to describe the role of care coordinator nationally and internationally. Although the core elements of the role are often presented in a similar fashion, the absence of definitions and a lack of comprehensive, standardised job descriptions has led to a lack of clarity within the literature. Despite the variability of quality in relation to the levels of evidentiary support for studies, overall there was a consistency as to the extent of contextual variables that influence the role, suggesting that the findings are plausible.

## Conclusion

The volume of articles identified as a result of the initial keyword search is illustrative of the extent to which research has been undertaken to improve the services and care provided for children with complex care needs. The literature highlights, however, that the role of care coordinator is one aspect of research that requires further investigation. Despite evidence that suggests that the role is pivotal in ensuring that care needs are sustained, the inconsistency across the literature highlights that much more needs to be done to improve services for this population if they are to continue being cared for in the community. The very nature of their complex needs means that their care is provided by a wide range of services, incorporating health, education, social and voluntary sectors, the fragmented nature of which demands effective care coordination is in place.
